# Associations of Quantitative and Qualitative Muscle Parameters With Second Hip Fracture Risk in Older Women: A Prospective Cohort Study

**DOI:** 10.1002/jbm4.10834

**Published:** 2023-10-25

**Authors:** Wenshuang Zhang, Yufeng Ge, Yandong Liu, Yi Yuan, Jian Geng, Fengyun Zhou, Pengju Huang, Jia Shi, Kangkang Ma, Zitong Cheng, Glen M. Blake, Minghui Yang, Xinbao Wu, Xiaoguang Cheng, Ling Wang

**Affiliations:** ^1^ Department of Radiology, Beijing Jishuitan Hospital Capital Medical University Beijing China; ^2^ Department of Radiology Peking University Fourth School of Clinical Medicine Beijing China; ^3^ Department of Orthopaedics and Traumatology, Beijing Jishuitan Hospital Capital Medical University Beijing China; ^4^ Beijing Key Laboratory of Mental Disorders, National Clinical Research Center for Mental Disorders & National Center for Mental Disorders, Beijing Anding Hospital Capital Medical University Beijing China; ^5^ National Institute for Nutrition and Health Chinese Center for Disease Control and Prevention Beijing China; ^6^ School of Biomedical Engineering & Imaging Sciences, King's College London St Thomas' Hospital London UK; ^7^ Sarcopenia Research Center, Beijing Research Institute of Traumatology and Orthopaedics, Beijing Jishuitan Hospital, National Center for Orthopaedics Fourth Clinical Medical College of Peking University Beijing China

**Keywords:** ELDERLY FEMALES, GOUTALLIER CLASSIFICATION, MUSCLE DENSITY, RISK PREDICTOR, SECOND HIP FRACTURE

## Abstract

Older women with a first hip fracture exhibit heightened susceptibility and incidence of second fracture and potentially severe consequences. This prospective study was to compare the predictive power of qualitative and quantitative muscle parameters for a second hip fracture in older women with a first hip fracture. A total of 206 subjects were recruited from the longitudinal Chinese Second Hip Fracture Evaluation study. Hip computed tomography (CT) scans were obtained immediately after the first fracture. Muscle fat infiltration was assessed according to the Goutallier classification qualitatively. Quantitative parameters included cross‐sectional area and density of gluteus maximus (G.MaxM) and gluteus medius and minimus (G.Med/MinM) muscles. CT X‐ray absorptiometry was used to measure the areal bone mineral density (aBMD) of the contralateral femur. Cox proportional hazards models were used to compute hazard ratios (HR) of second hip fracture risk. The mean age of subjects was 74.9 (±9.5) years at baseline. After 4.5 years, 35 had a second hip fracture, 153 without a second hip fracture, and 18 died. Except for the combined G.MinM Goutallier grade 3 and 4 groups before adjustment for covariates (HR = 5.83; 95% confidence interval [CI] 1.49–22.83), there were no significant HRs for qualitative classification to predict a second hip fracture. Among quantitative metrics, after adjustment for covariates, G.Med/MinM density was significant in the original (HR = 1.44; CI 1.02–2.04) and competing risk analyses (HR = 1.46; CI 1.02–2.07). After additional adjustment for femoral neck (FN) aBMD, G.Med/MinM density remained borderline significant for predicting a second hip fracture in competing risk analysis (HR = 1.43; CI 0.99–2.06; *p* = 0.057). Our study revealed that Goutallier classification was less effective than quantitative muscle metrics for predicting hip second fracture in this elderly female cohort. After adjustment for FN aBMD, G.Med/MinM density is a borderline independent predictor of second hip fracture risk. © 2023 The Authors. *JBMR Plus* published by Wiley Periodicals LLC on behalf of American Society for Bone and Mineral Research.

## Graphical abstract

The Goutallier classification was less effective than quantitative muscle metrics associated with second hip fracture risk in the female low‐energy hip fracture cohort. After adjustment for FN BMD, G.Med/MinM density was a borderline independent predictor for a second hip fracture in elderly females with an initial hip fracture. These findings provide valuable insights into the critical role of integrating muscle quantitative assessments into routine clinical evaluations.

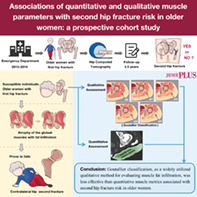

## Introduction

Hip fractures and subsequent second fractures are associated with increased morbidity, mortality, and substantial health care expenditure.^(^ ^1^, ^2^, ^3^, ^4^ ^)^ The risk of a second hip fracture is notably elevated among individuals who have already experienced an initial hip fracture, particularly among elderly females, who experience higher incidence rates and greater detrimental effects.^(^ ^5^, ^6^, ^7^ ^)^ Given the heightened vulnerability of older women to primary and secondary hip fractures, it becomes imperative to place greater emphasis on the identification and assessment of the risk of secondary fractures in this population. Notably, the occurrence of a second hip fracture in elderly females presents a higher risk of prolonged immobility and detrimental effects compared with the first fracture.^(^ ^5^ ^)^ Therefore, improvement in predicting the risk of a second hip fracture, particularly in high‐risk individuals such as elderly women, is of paramount importance for the implementation of targeted preventive strategies.^(^ ^8^ ^)^

Muscle strength, particularly in the hip and thigh muscles, plays a critical role in maintaining stability during activities such as walking, standing, and changing direction.^(^ ^9^ ^)^ Muscle weakness, especially in the lower extremities, compromises balance and contributes to frailty and the risk of falls, leading to hip fracture and loss of the ability to live independently. Impaired postural control and a diminished ability to recover from perturbations contribute to an elevated risk of falls that may result in subsequent hip fractures.^(^ ^5^, ^6^, ^10^ ^)^ Impaired muscle function contributes to reduced muscle performance and further exacerbation of fatty infiltration.^(^ ^11^, ^12^ ^)^

Muscle fat infiltration (MFI) can be evaluated radiologically using both qualitative and quantitative methods.^(^ ^13^, ^14^ ^)^ The Goutallier classification (GC)^(^ ^15^ ^)^ is a widely utilized qualitative grading system developed for assessing MFI in clinical practice because of its convenience and ease of operation, primarily in the context of musculoskeletal imaging. This classification system provides a feasible approach to qualitatively evaluating the severity of adipose tissue infiltration within a specific muscle or muscle group.^(^ ^15^ ^)^ Furthermore, in a previous study, we demonstrated the predictive ability of quantitative muscle parameters, particularly muscle density, for assessing the risk of a subsequent hip fracture in a cohort that comprised both male and female participants who had suffered an initial fracture.^(^ ^16^ ^)^ However, among elderly females, the specific role of quantitative muscle parameters in predicting the risk of a second hip fracture has not been investigated, especially the comparison of the quantitative and qualitative muscle parameters in risk prediction.

Therefore, we hypothesized that the Goutallier classification may substitute for quantitative muscle parameters like muscle density as a time‐saving qualitative evaluation tool for predicting the risk of second hip fractures. In this study, our primary aim was to investigate the predictive value of both qualitative and quantitative muscle parameters in assessing the risk of a second hip fracture among elderly females who had experienced a first fracture and, additionally, to make a comparison between them. Secondly, we sought to determine whether muscle quantitative parameters independently predict the risk of female second hip fractures, irrespective of hip bone mineral density (BMD).

## Materials and Methods

### Study population

This investigation examined the female patients who participated in a prospective longitudinal study, the Chinese Second Hip Fracture Evaluation (CSHFE) trial (ClinicalTrials.gov Identifier: NCT03461237).^(^ ^17^ ^)^ The institutional review board of our hospital approved the study, and all participants provided written informed consent. Patients enrolled in the CSHFE trial sought medical care at the emergency department of our hospital because of a low‐energy hip fracture during the period from May 2015 to June 2016. Females participating in the CSHFE trial were followed up for a median duration of 4.5 years, spanning from 2015 to 2016 until 2019 to 2020. We have previously published the clinical approach and the inclusion and exclusion criteria utilized in the study.^(^ ^16^, ^18^ ^)^ In summary, the study included only fully ambulatory, community‐dwelling Chinese Han adults before the primary hip fracture. Exclusions were those who could not sit and stand independently, walk without assistance, or had pain preventing testing. Additional criteria for exclusion encompassed stroke, neurologic and metabolic disorders, rheumatic diseases, heart failure, severe chronic obstructive pulmonary disease, coagulation disorders, and other functional limitations. Notably, the inclusion criteria for a frailty hip fracture specifically focused on falls from standing or sitting height as the primary cause as opposed to falls from higher elevations or traumatic incidents with significant impact force. Female patients who were still alive at the end of the follow‐up period were divided into two groups: those who had experienced only a single hip fracture and those who suffered a second hip fracture during the follow‐up period.

### CT examinations

CT scans of the baseline visit obtained within 48 hours after the occurrence of the first fragility hip fracture were utilized for the purpose of the following analyses. All subjects underwent hip scans on one of two Toshiba Aquilion CT scanners (Toshiba Medical Systems Division, Tokyo, Japan) with a spiral CT technique. Acquisition was performed in the supine position, encompassing the region from the superior aspect of the acetabulum to 3 cm distal to the lesser trochanter. Scan parameters were 120 kVp; 125 mAs; field of view, 500 mm; matrix, 512 × 512; reconstructed slice thickness, 1 mm.

### Qualitative visual MFI evaluation

CT images were independently graded based on a Goutallier classification by two radiologists (Reader 1, with 6 years of experience, and Reader 2, with 5 years of experience) as grade 0 (no fatty streaks), grade 1 (some fatty streaks), grade 2 (more muscle than fat), grade 3 (as much fat as muscle), and grade 4 (less muscle than fat).^(^ ^15^ ^)^ Any disagreement was resolved by discussion and consensus, including a third more experienced reader (Reader 3, with 11 years of experience). The final agreed classifications were formulated and available for further analysis. The slices selected for qualitative evaluation corresponded to the level of the greater trochanter for the gluteus maximus (G.Max) and the level of the third sacral vertebra (S3) for the gluteus medius (G.Med) and minimus (G.Min) muscles, which aligned with the regions assessed quantitatively.

### Quantitative muscle density and bone density assessments

For the non‐fractured hips of participants, we measured the cross‐sectional density and area of the G.Max and the gluteus medius and minimus (G.Med/Min) muscles by using OsiriX software (Lite Version 10.0.2, Pixmeo, Geneva, Switzerland). Regions of interest (ROIs) were selected at the level of the greater trochanter for G.Max and at the S3 level for G.Med/Min. The ROI for G.Max was manually delineated following the outline of the gluteus maximus. The gluteus medius and minimus were both outlined as a whole, including the fascia between the two muscles. The computed tomography X‐ray absorptiometry technique (Version 4.2.3, Mindways Inc., Austin, TX, USA) was used to calculate the areal bone mineral density (aBMD, g/cm^2^) of the femoral neck (FN), total hip (TH), intertrochanter (IT), and trochanter (TR) regions from the acquired hip CT scans. The measurement diagrams and pertinent details have been documented in two preceding articles.^(^ ^16^, ^17^ ^)^

### Clinical data collection

Comprehensive baseline clinical data were recorded, including essential demographic and health‐related information. These parameters comprised age, sex, weight, height, body mass index (BMI), Parker Mobility Score (PMS), blood pressure, presence of hypertension, history of previous fractures, osteoarthritis, coronary heart disease (CHD), type 2 diabetes mellitus (T2DM), and preventive treatment for osteoporosis. By capturing this wide array of clinical variables, a detailed profile of the participants was established, enabling a thorough examination of their baseline characteristics and their potential influence on the outcomes of interest.

The PMS is a well‐established measurement tool for evaluating mobility that provides a quantitative measure to assess and track individual mobility over time, covering a scale of 0 to 9 points in total, with higher scores reflecting better mobility and independence in performing three specific tasks: walking within the house, walking out of the residence, and engaging in shopping activities. Scoring criteria for each functional task are based on the level of difficulty experienced by the patient: unimpeded mobility (3 points), mobility aided by a walking stick or similar assistive devices (2 points), mobility with assistance from another individual (1 point), or complete inability (0 points).^(^ ^19^ ^)^ The evaluator assesses the performance of participants based on predefined criteria and assigns a score accordingly. To ensure reliability and consistency, the evaluation process should be conducted by trained orthopedists following standardized protocols for administering the PMS. PMS was evaluated within a 3‐month window before the first hip fracture, second fracture, death, and telephone interview for patients without a second hip fracture. Only subjects with a PMS >3 points were involved in the subsequent statistical analysis.

### Statistical analysis

Continuous variables were expressed as means ± standard deviation, and categorical variables were expressed as numbers and percentages. Normality of the continuous variables was estimated by the Shapiro–Wilk test. Two‐sample comparisons were performed using *t* tests or Wilcoxon rank sum tests for continuous variables and chi‐square tests for categorical variables. A kappa analysis was performed to determine the interobserver reliability of qualitative Goutallier grades. Traditional Cox proportional hazards models were used to compute hazard ratios (HR) of second hip fracture risk in subjects with a first hip fracture. Furthermore, to account for competing risks, cause‐specific hazard models were performed, taking into account deaths as a competing risk that occurred without the incidence of a second fracture. The Statistical Analysis System (SAS 9.4 for Windows; SAS Institute Inc., Cary, NC, USA) was used for all statistical analyses.

## Results

### Baseline characteristics of the study population

**Fig. 1:**
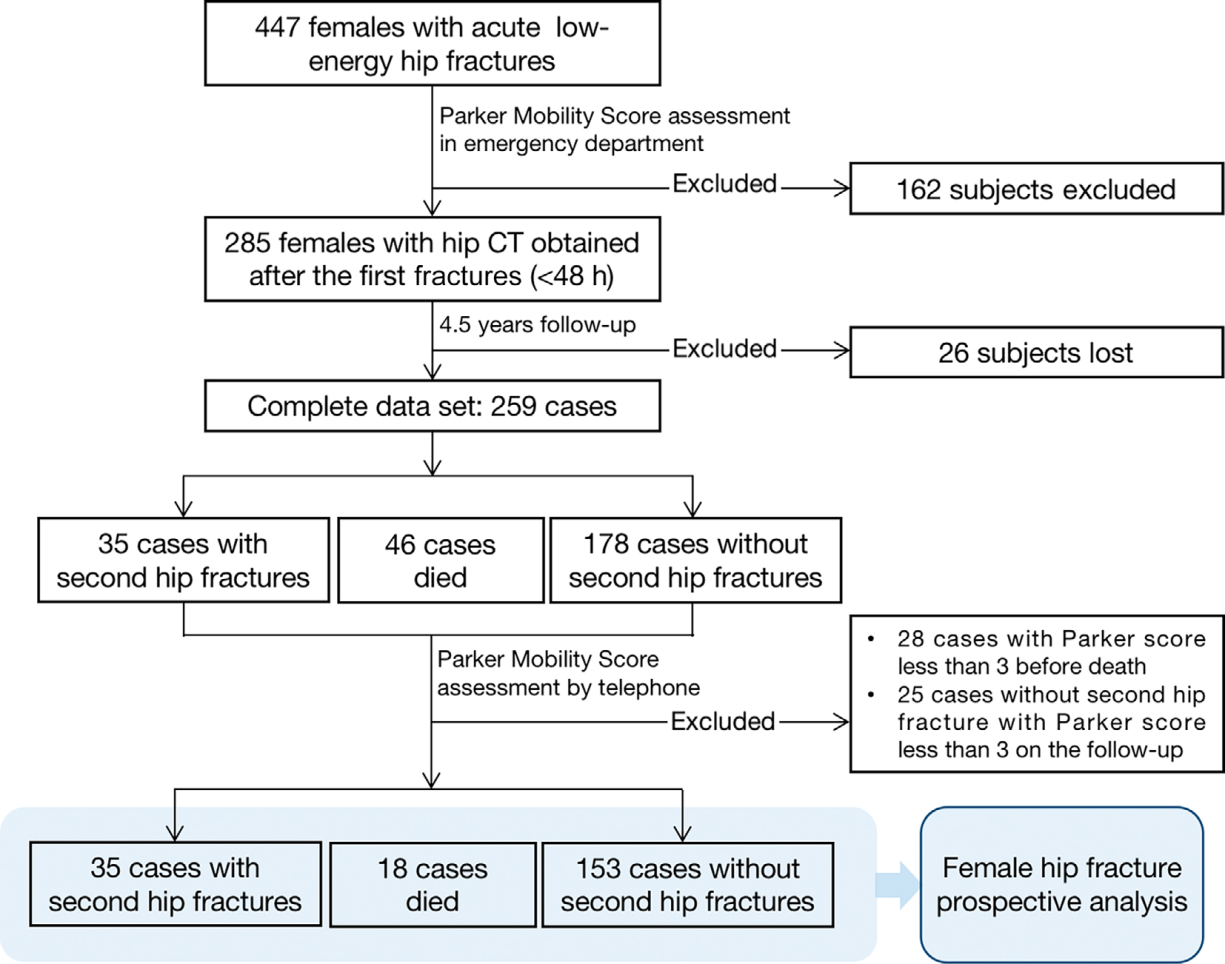
Flow chart of participant selection for the study.

Figure 1 presents a flow chart of the female participants enrolled in the study. Subjects who exhibited a PMS less than 3 during postoperative evaluation or before their death were excluded from the study, ensuring that the analysis focused on individuals with at least some mobility. After 4.5 years of follow‐up, 28 cases were excluded before their deaths and 25 without a second hip fracture. Finally, 35 elderly females with a second hip fracture, 18 females who died, and 153 cases without a second hip fracture remained. The baseline characteristics of these participants are shown in Table 1. The females who experienced a second fracture or mortality were found to be older and have higher PMS at the end of follow‐up in comparison to the surviving non‐refracture group.

**Table 1:** General Characteristics

	Female hip fracture patients				
	Died	Refracture	Non‐refracture	*p* Value^a^	
Characteristics (mean ± SD)	(1)	(2)	(3)	(2) vs. (3)	(2) vs. (1)+ (3)
Sample size	18	35	153		
Age (years)	79.53 ± 8.13	79.33 ± 7.70	73.41 ± 9.56	<0.001	<0.001
HA, % (*n*)	22.2 (4)	45.7 (16)	37.9 (58)	0.39	0.29
FN fractures, % (*n*)	38.9 (7)	45.7 (16)	57.5 (88)	0.21	0.29
Antiosteoporosis treatment, % (*n*)	16.7 (3)	20 (7)	17 (26)	0.67	0.67
Height (cm)	158.50 ± 5.54	158.33 ± 4.68	158.50 ± 8.20	0.92	0.92
Weight (kg)	54.47 ± 10.53	58.13 ± 10.67	58.62 ± 9.91	0.82	0.99
BMI (kg/m^2^)	21.61 ± 3.71	23.75 ± 4.6	23.41 ± 5.23	0.73	0.58
Parker Mobility Score 1	8.17 ± 1.04	8.34 ± 1.21	8.67 ± 0.89	0.14	0.21
Parker Mobility Score 2	6.00 ± 1.33	6.64 ± 2.36	7.88 ± 1.32	0.01	0.02
SBP (mmHg)	146.00 ± 20.02	145.74 ± 16.91	143.07 ± 20.85	0.48	0.53
DBP (mmHg)	77.47 ± 11.48	76.03 ± 11.46	75.61 ± 11.78	0.85	0.92
Hypertension, % (*n*)	11.1 (2)	28.6 (10)	25.5 (39)	0.71	0.57
T2DM, % (*n*)	61.1 (11)	34.3 (12)	51.6 (79)	0.06	0.048
History of CHD, % (*n*)	0 (0)	2.9 (1)	2 (3)	0.57	0.53
Previous fractures, % (*n*)	11.1 (2)	20 (7)	30.1 (46)	0.23	0.33
OA, % (*n*)	11.1 (2)	5.7 (2)	11.1 (17)	0.54	0.54
G.MaxM area (cm^2^)	29.50 ± 4.34	28.10 ± 5.76	30.81 ± 6.44	0.01	0.04
G.MaxM density (HU)	20.64 ± 7.97	20.30 ± 7.86	23.61 ± 6.82	0.01	0.02
G.Med/MinM density (HU)	28.12 ± 9.29	27.49 ± 5.23	31.03 ± 6.26	<0.001	<0.001
TH aBMD (g/cm^2^)	0.54 ± 0.14	0.50 ± 0.10	0.57 ± 0.12	<0.001	0.002
FN aBMD (g/cm^2^)	0.45 ± 0.09	0.47 ± 0.14	0.49 ± 0.11	0.09	0.13
TR aBMD (g/cm^2^)	0.35 ± 0.10	0.33 ± 0.09	0.38 ± 0.09	0.005	0.008
IT aBMD (g/cm^2^)	0.68 ± 0.18	0.61 ± 0.11	0.70 ± 0.14	<0.001	<0.001
G.MaxM G grades, grade (*n*)				0.13	0.146
	1 (7)	1 (12)	1 (74)		
	2 (11)	2 (22)	2 (78)		
		3 (1)	3 (1)		
G.MedM G grades, grade (*n*)				0.112	0.145
	1 (5)	1 (15)	1 (89)		
	2 (13)	2 (19)	2 (63)		
		3 (1)	3 (1)		
G.MinM G grades, grade (*n*)				0.054	0.138
	2 (13)	1 (3)	1 (35)		
	3 (5)	2 (23)	2 (98)		
		3 (8)	3 (14)		
		4 (1)	4 (6)		

*Note*: Parker Mobility Score 1: assessment obtained before first hip fracture surgery; Parker Mobility Score 2: refracture group: assessment within 3 months before second hip fracture; death group: assessment within 3 months before death; group without second fracture: assessment of mobility before follow‐up visit.

Abbreviations: BMI = body mass index; CHD = coronary heart diseases; DBP = diastolic blood pressure; FN = femoral neck; G grades = Goutallier grades; G.MaxM = gluteus maximus muscle; G.MedM = gluteus medius muscle; G.MinM = gluteus minimus muscle; HA = hip arthroplasty (including total hip arthroplasty and hemiarthroplasty); IT = intertrochanter; OA = osteoarthritis; SBP = systolic blood pressure; T2DM = type 2 diabetes; TH = total hip; TR = trochanter.

^a^ The *p* value was obtained using chi‐square tests for categorical variables and two‐sample Wilcoxon tests for continuous variables.

### Qualitative assessment of muscle fat infiltration

**Fig. 2:**
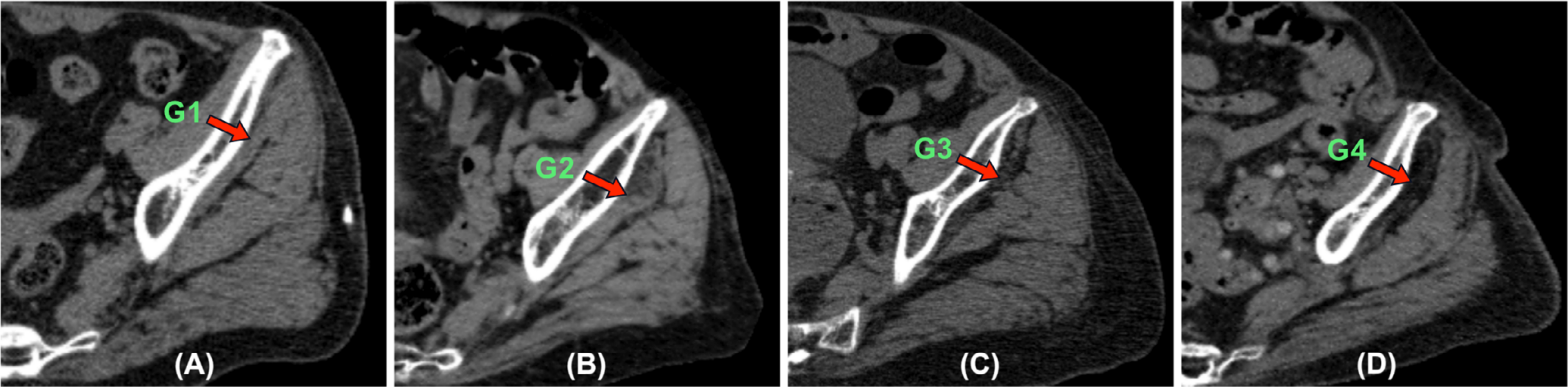
Assessment of gluteus muscle fat infiltration using Goutallier classification on axial CT images, illustrated with gluteus minimus muscle (*A*–*D*). (*A*) Grade 1 (G1), indicating some fatty streaks within muscle. (*B*) Grade 2 (G2), indicating less fat compared with the muscle tissue. (*C*) Grade 3 (G3), with fat content similar to that of the muscle tissue. (*D*) Grade 4 (G4), with a higher fat content than the gluteus minimus muscle tissue.

Figure 2 illustrates several degrees of fat infiltration in gluteal minimus muscle using the Goutallier classification system. The results of Goutallier grades (G grades) for G.Max, G.Med, and G.Min muscle in three subgroups (died, refracture, and non‐refracture) are shown in Table 1. The qualitative evaluation of MFI for G.Max was G grades 1, 2, and 3 in 34.3%, 62.8%, and 2.9% of participants in the refracture group, and in 48.4%, 51.0%, and 0.6%, respectively, in the non‐refracture group. For G.Med, MFI qualitative evaluation was G grades 1, 2, and 3 in 42.8%, 54.3%, and 2.9% in the refracture group, and 58.2%, 41.2%, and 0.6%, respectively, in the non‐refracture group. Furthermore, evaluation for G.Min was grades 1, 2, 3, and 4 in 8.6%, 65.7%, 22.8%, and 2.9% in the refracture group, and 22.9%, 64.0%, 9.2%, and 3.9%, respectively, in the non‐refracture group. There were no significant differences statistically in G grades of G.Max, G.Med, or G.Min muscle between the refracture and non‐refracture groups. In addition, moderate interobserver variability was shown with a *k* value of 0.53.

### Quantitative and qualitative muscle parameters: prediction of second hip fracture

**Fig. 3:**
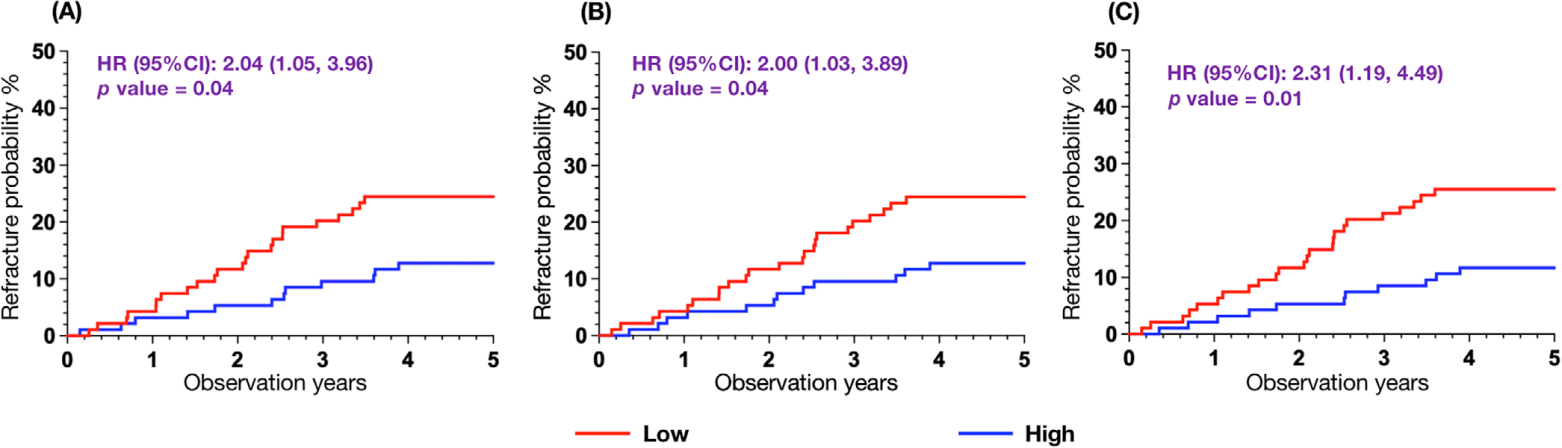
Kaplan–Meier curves for probability of second hip fracture by low versus high parameter values using median as cut‐points. (*A*) Gluteus maximus (G.MaxM) area, (*B*) G.MaxM density, (*C*) gluteus medius and minimus (G.Med/MinM) density. CI = confidence interval; HR = hazard ratio.

Figure 3 depicts the cumulative incidence of a second hip fracture using Kaplan–Meier survival curves. The plots demonstrate the differentiation of high‐ and low‐risk groups based on the median values of each parameter. Notably, lower G.MaxM area and lower densities of G.MaxM and G.Med/MinM were associated with a higher probability of a second hip fracture. In the traditional unadjusted Cox proportional hazards model, all quantitative muscle parameters exhibited statistical significance in relation to the risk of a second hip fracture. However, after adjustments for age, T2DM, and PMS obtained before surgery, only G.Med/MinM density remained significantly associated with higher refracture risks both in the original Cox proportional hazards model (HR = 1.44; 95% confidence interval [CI] 1.02–2.04; *p* = 0.03) and the competing risk analysis (HR = 1.46; 95% CI 1.02–2.07; *p* = 0.04) (Table 2).

**Table 2:** Hazard Ratios of Quantitative Muscle Parameters in Per SD Decrease and Qualitative Muscle Parameters for Risk of Second Fracture

	Original analyses (35 vs. 153)				Competing risk analyses^a^ (35 vs. 171)			
	Unadjusted		Adjusted^b^		Unadjusted		Adjusted^b^	
Muscle parameters	HR (95% CI)	*p* Value	HR (95% CI)	*p* Value	HR (95% CI)	*p* Value	HR (95% CI)	*p* Value
G.MaxM area (cm^2^)	1.52 (1.07, 2.17)	0.02	1.15 (0.78, 1.68)	0.49	1.46 (1.02, 2.09)	0.04	1.15 (0.75, 1.76)	0.52
G.MaxM density (HU)	1.61 (1.07, 2.40)	0.02	1.26 (0.78, 2.03)	0.35	1.57 (1.11, 2.24)	0.01	1.30 (0.87, 1.94)	0.20
G.Med/MinM density (HU)	1.49 (1.14, 1.93)	<0.01	1.44 (1.02, 2.04)	0.03	1.40 (1.12, 1.76)	<0.01	1.46 (1.02, 2.07)	0.04
G.MaxM G grades								
G1	1 [Reference]		1 [Reference]		1 [Reference]		1 [Reference]	
G2 + G3	1.71 (0.85, 3.41)	0.13	1.51 (0.69, 3.32)	0.30	1.68 (0.83, 3.37)	0.15	1.62 (0.74, 3.57)	0.23
G.MedM G grades								
G1	1 [Reference]		1 [Reference]		1 [Reference]		1 [Reference]	
G2 + G3	1.79 (0.92, 3.47)	0.09	1.15 (0.48, 2.76)	0.76	1.68 (0.86, 3.28)	0.13	1.18 (0.51, 2.71)	0.70
G.MinM G grades								
G1	1 [Reference]		1 [Reference]		1 [Reference]		1 [Reference]	
G2	2.50 (0.74, 8.38)	0.14	2.02 (0.54, 7.50)	0.30	2.01 (0.72, 7.95)	00.16	1.99 (0.52, 7.54)	0.31
G3 + G4	4.70 (1.24, 17.83)	0.02	2.73 (0.54, 13.89)	0.23	4.34 (1.09, 14.83)	0.04	2.49 (0.53, 11.64)	0.25

Abbreviations: G grades = Goutallier grades; G.MaxM = gluteus maximus muscle; G.MedM = gluteus medius muscle; G.MinM = gluteus minimus muscle; HR = hazard ratio; CI = confidence interval.

^a^ As for refracture risk, we did the competing risk analyses using cause‐specific hazard models given that total deaths (*n* = 18) occurring in the absence of refracture events are the competing risks.

^b^ Adjusted for age, type 2 diabetes, and Parker Mobility Score before first hip fracture surgery.

**Fig. 4:**
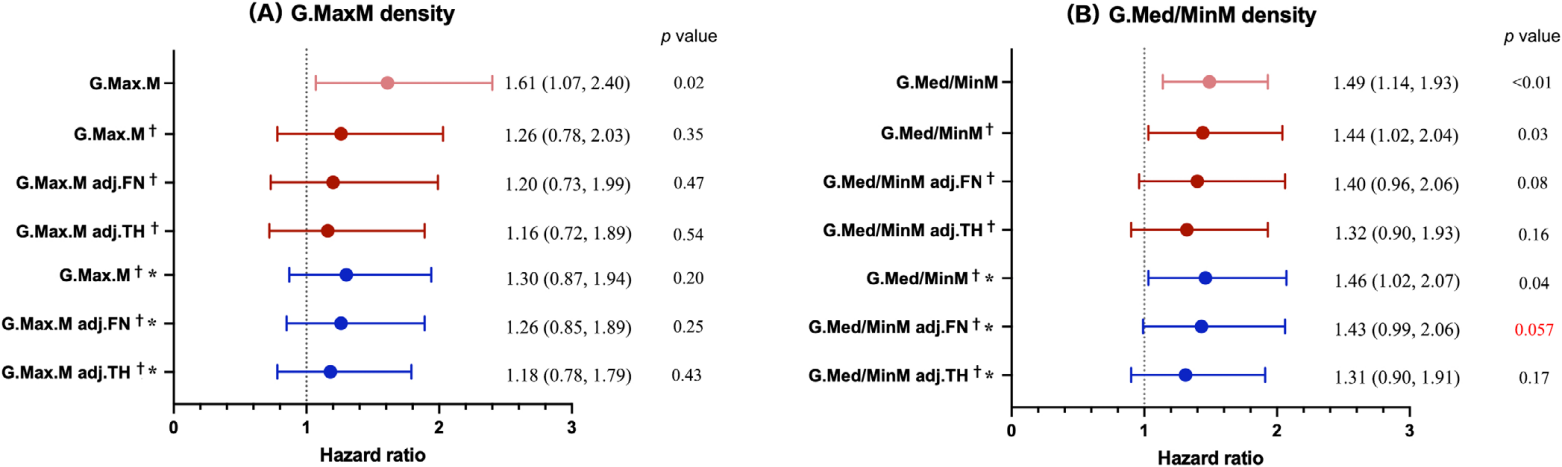
Unadjusted and adjusted hazard ratios of second hip fracture per one SD increase of gluteus maximus (G.MaxM) density (*A*) and gluteus medius and minimus (G.Med/MinM) density (*B*). Adj.FN refers to adjustment for femoral neck (FN) areal bone mineral density (aBMD); adj.TH refers to adjustment for total hip (TH) aBMD. ^†^Adjusted for age, type 2 diabetes, and Parker Mobility Score before first hip fracture surgery; *Competing risk analyses.

In all models examined, the qualitative muscle parameters G.MaxM and G.MedM G grades were found to be statistically nonsignificant (Table 2). The significance of the combined G.MinM G3 and G4 grades in predicting the risk of hip refractures was initially confirmed through unadjusted models in both the original (HR = 4.70; 95% CI 1.24–17.83; *p* = 0.02) and competing risk analyses (HR = 4.34; 95% CI 1.09–14.83; *p* = 0.04). However, the association was found to be statistically insignificant after adjusting for covariates in both the original (HR = 2.73; 95% CI, 0.54–13.89; *p* = 0.23) and competing risk analyses (HR = 2.49; 95% CI 0.53–11.64; *p* = 0.25) (Table 2). For G.Med/MinM density, the HR barely changed after adjustment for FN aBMD (HR = 1.50; 95% CI 1.16–1.94). However, G.Med/MinM density remained borderline statistically significant for predicting the risk of a second hip fracture after adjustment for FN aBMD and other covariates after the competing risk analysis (HR = 1.43; CI 0.99–2.06; *p* = 0.057) (Fig. 4).

## Discussion

To our knowledge, this is the first prospective study comparing the strength of qualitative and quantitative muscle parameters for predicting the risk of a second hip fracture in elderly females. Fractures in postmenopausal women are strongly correlated with an increased risk of subsequent fractures, which holds pivotal implications for clinicians in terms of assessing fall risks and predicting second hip fractures after initial fractures. It is crucial to emphasize that osteoporotic hip refractures are commonly associated with falls, and the risk of falls is influenced, in part, by muscle function and quality. Muscle quality assessment in CT can be conducted using the qualitative Goutallier classification system or by measuring quantitative muscle parameters. In this prospective study of females, the predictive performance of the qualitative method, the Goutallier classification, was found to be unsatisfactory and was less effective than quantitative muscle metrics for predicting hip refractures. Conversely, the quantitative parameters, particularly muscle density, exhibited superior performance. The increased risk of a second hip fracture after an initial fracture was highest in individuals with more severe fat infiltration of muscle, specifically women with lower G.Med/MinM density.

The Goutallier classification serves as a widely used tool for evaluating fatty degeneration or infiltration of musculature,^(^ ^20^, ^21^ ^)^ particularly in conditions such as rotator cuff tears^(^ ^22^, ^23^ ^)^ and muscle atrophy.^(^ ^24^, ^25^ ^)^ Previous studies have shown the prognostic value of the Goutallier classification in clinical and radiologic outcome studies on the fatty infiltration of the rotator cuff musculature.^(^ ^22^, ^26^, ^27^ ^)^ Engelken and colleagues reported that the diagnostic performance of the Goutallier classification is good for quantifying fatty infiltration of the gluteus muscles.^(^ ^28^ ^)^ Wu and colleagues reported that visual dual‐energy CT assessment using the Goutallier classification could distinguish severe from normal and moderate fat infiltration of lumbar paravertebral muscles in subjects with low back pain.^(^ ^13^ ^)^ Several prior studies have reported that the Goutallier classification correlated strongly with quantitative measurements^(^ ^29^, ^30^ ^)^ and that the Goutallier classification and quantitative measures had comparable diagnostic efficiency without a statistically significant difference.^(^ ^28^, ^31^ ^)^ Despite the widespread use and diagnostic value of the Goutallier classification system, there is currently no study comparing its effectiveness in predicting the risk of hip refracture to that of quantitative muscle parameters. Additionally, the role of the Goutallier classification in clinical practice remains a subject of considerable controversy.

In the present study, the qualitative Goutallier classification was compared with quantitative muscle parameters as risk predictors of a second hip fracture, and the results demonstrated that the Goutallier classification system was not effective in predicting secondary hip fractures among females with initial hip fractures. Likewise, a previous study of lumbar paraspinal muscle reported that the lumbar lordosis could not be predicted by the evaluation of muscle quality using the Goutallier classification.^(^ ^32^ ^)^ The poor predictive performance mentioned above may in part be attributed to inconsistencies in the Goutallier classification. Some researchers have questioned its validity because of its moderate (*k* value range, 0.41–0.60) or even fair (*k* value range, 0.21–0.40) inter‐ and intra‐observer reliability.^(^ ^22^, ^33^, ^34^ ^)^ Furthermore, the previous study demonstrated that increased clinical experience or a longer duration of clinical practice does not necessarily enhance intra‐observer reliability.^(^ ^33^ ^)^ Slabaugh and colleagues observed a negative correlation between years of practice and the level of agreement in the application of the Goutallier classification for evaluating fatty infiltration of the rotator cuff.^(^ ^33^ ^)^ In our study, the interobserver reliability among two young clinicians was also moderate, and the final Goutallier classification results were determined through consultation with an experienced senior doctor. Considering the aforementioned factors, the steps involved in determining the final Goutallier grades in this study could not fully guarantee an accurate reflection of the extent of muscle fat infiltration. The subjectivity of the Goutallier classification may have contributed to the suboptimal predictive performance observed. Clinicians visually assessed the degree of fatty infiltration in affected muscles, which is manifested by regions of reduced radiodensity commonly observed in noncontrast CT scans. Based on clinical experience, clinicians have observed that differentiating between grades 2 and 3 poses greater challenges than differentiating between grades 3 and 4. In this study, the final classification of the Goutallier grades for the gluteus minimus was primarily concentrated in grades 1 to 3. In the Cox model analysis, because of the relatively low number of subjects with grade 4 for the gluteus minimus, the subjects with grades 3 and 4 were combined into one group. It is worth noting that misclassification between grades 2 and 3 might potentially impact the predictive performance of the gluteus minimus muscle. The lack of standardized criteria and clear thresholds makes it challenging to establish a definitive relationship between Goutallier grades and the risk of a second hip fracture.

Among the quantitative muscle parameters, only G.Med/MinM density exhibited sustained statistical efficacy in predicting the risk of subsequent hip fractures in older women after adjusting for covariates. This notable effectiveness was observed not only in the original Cox model analysis but also in the competing risk model, highlighting the robustness and reliability of G.Med/MinM density as a predictive factor among females. This result is in accordance with the conclusions of prior investigations conducted by Wang and colleagues.^(^ ^35^ ^)^ This finding may signify that when gluteal muscle degeneration occurs, the gluteus minimus and medius muscles exhibit heightened susceptibility to fat infiltration, and the reduction in muscle density may emerge earlier and be more pronounced.^(^ ^9^, ^36^ ^)^ Kiyoshige and colleagues reported that fatty degeneration of the gluteus minimus muscle could be a predictor of falls in elderly people.^(^ ^37^ ^)^ Muscle density provides valuable information about the accumulation of both intermuscular adipose tissue and intramyocellular lipids, which are associated with muscle dysfunction, decreased strength, and impaired mobility.^(^ ^14^, ^38^ ^)^ Thus, elderly females with an initial hip fracture accompanied by lower muscle density are more likely to experience reduced muscle function and increased vulnerability to falls, leading to a higher risk of a further hip fracture. Furthermore, muscle density provides quantitative and objective measurements that can be easily obtained through imaging techniques such as CT scans. These measurements offer a standardized and reliable assessment of muscle quality, allowing for more accurate risk stratification and prediction of the risk of a second hip fracture.

Interestingly, G.Med/MinM density exhibited borderline significance after an additional adjustment for FN aBMD in the competing risk analysis with a hazard ratio of 1.43, different from the insignificant result in the original risk model with a hazard ratio of 1.40. To some extent, this indicates that non‐hip refracture‐related deaths during the follow‐up period serve as competing factors influencing the risk of subsequent hip fractures in elderly women who have experienced an initial hip fracture. Nevertheless, the same pattern mentioned above was not observed for total hip aBMD. This discrepancy differs from our previous research conducted on the general population without sex stratification, in which G.Med/MinM density remained significant after an additional adjustment for both FN and TH aBMD,^(^ ^16^ ^)^ suggesting a certain level of specificity within the female cohort.

Our findings underscore the necessity of integrating quantitative muscle assessments into regular clinical evaluations. The quantitative muscle parameters, especially G.Med/MinM density, could offer a clearer, objective perspective on musculoskeletal health and associated risks of hip second fractures. In clinical contexts, precise quantitative measurements enable the implementation of individualized therapeutic strategies. Should the muscle metrics reveal increased risks of hip second fracture, specific therapeutic exercises may be advised to rectify identified muscle deficiencies. The broader practical implications are not just understanding the muscle‐health and recurrent hip fracture correlation but also championing holistic musculoskeletal health, emphasizing focused rehabilitation, and prioritizing preventative measures to elevate the quality of life for elderly females.

The innovative aspect of our study was the comparison of qualitative Goutallier classifications and quantitative cross‐sectional muscle parameters for predicting the risk of second hip fractures among elderly females with an initial hip fracture. The strengths of our study are that all females analyzed had low‐energy fractures, and all CT images were obtained in the first 48 hours after a hip fracture to minimize fracture‐related changes in muscles and other tissues. Furthermore, the Goutallier grades were determined by three trained clinicians through a rigorous assessment process, ensuring a reliable outcome of the qualitative grading results.

This study has several limitations. First, there was an uneven distribution of Goutallier grades among the different gluteal muscles, with a relatively low number of cases classified as grades 3 and 4 in the gluteus maximus and medius muscles and a scarcity of grade 4 cases in the gluteus minimus muscle. Second, the gluteus medius and minimus muscle area (G.Med/MinM area) was not included in the analysis because of the limited coverage of the S3 level in some CT scans. Consequently, the measurements of the G.Med/MinM area were measured at the S4 or S5 levels. Furthermore, prior investigations have substantiated that obtaining measurements for the G.Med/MinM area at the S4 or S5 levels can introduce a potential bias of up to 10% when compared with the measurements taken at the S3 level. Therefore, we made the decision not to incorporate the G.Med/MinM area in our analysis. Volumetric measurements of muscles may improve this measurement bias.^(^ ^39^ ^)^ Third, CT scans of the hip were performed at baseline and were not repeated, resulting in a lack of data on changes in the Goutallier grades of the gluteal muscles after the occurrence of the first hip fracture.

In conclusion, the Goutallier classification, as a widely utilized qualitative method for evaluating muscle fat infiltration, was less effective than quantitative muscle metrics associated with second hip fracture risk in the female low‐energy hip fracture cohort. Furthermore, after adjustment for FN BMD, G.Med/MinM density was a borderline independent predictor for a second hip fracture in elderly females with an initial hip fracture. These findings provide valuable insights into the critical role of integrating muscle quantitative assessments into routine clinical evaluations, thereby refining risk evaluation and guiding tailored therapeutic interventions, which hold promise for optimizing fracture prevention strategies, enhancing patient management, and potentially inspiring targeted therapeutic interventions to safeguard overall musculoskeletal health and even reduce recurrent hip fracture risk in the future.

## Author Contributions

**Wenshuang Zhang:** Data curation; formal analysis; methodology; writing – original draft; writing – review and editing. **Yufeng Ge:** Data curation; supervision; writing – review and editing. **Yandong Liu:** Data curation; investigation; visualization. **Yi Yuan:** Data curation; software; validation. **Jian Geng:** Data curation; writing – review and editing. **Fengyun Zhou:** Data curation. **Pengju Huang:** Data curation. **Jia Shi:** Methodology; visualization. **Kangkang Ma:** Data curation. **Zitong Cheng:** Data curation. **Glen M. Blake:** Supervision; writing – review and editing. **Minghui Yang:** Funding acquisition; investigation; resources; supervision. **Xinbao Wu:** Project administration; resources. **Xiaoguang Cheng:** Conceptualization; funding acquisition; investigation; project administration; resources; software; supervision; validation. **Ling Wang:** Conceptualization; formal analysis; funding acquisition; project administration; resources; supervision; writing – review and editing.

## Disclosures

The authors declare no conflicts of interest.

### Peer Review

The peer review history for this article is available at https://www.webofscience.com/api/gateway/wos/peer‐review/10.1002/jbm4.10834.

## Data Availability

The data for the Chinese Second Hip Fracture Evaluation (CSHFE) are not presently available to be shared. These data might be available at a future stage.
